# A Systematic Review of Help-Seeking Barriers for Racial-Ethnic Minority Caregivers Accessing Autism Diagnostic and Intervention Services

**DOI:** 10.1007/s40615-025-02555-x

**Published:** 2025-08-05

**Authors:** Amani Khalil, Jane Yatcilla, Niamh Christie, Xiang Zhou

**Affiliations:** 1 Department of Educational Studies, Purdue University, West Lafayette, IN 47907 USA; 2 Information Studies, Purdue University, West Lafayette, IN 47907 USA; 3 Department of Psychological Sciences, Purdue University, West Lafayette, IN 47907 USA

**Keywords:** Autism, Help-seeking barriers, Health disparities, Developmental disabilities, Racial minority parents, Developmental disabilities, Autism diagnosis, Autsim intervention

## Abstract

Caregivers play an essential role in early help-seeking and intervention for children with Autism Spectrum Disorder (ASD). Caregivers, therefore, provide a crucial role in helping to address the racial and ethnic disparity identified in accessing ASD intervention and diagnostic services (Bejarano-Martín et al., *Journal of Autism and Developmental Disorders 50*(9), 3380–3394, 2020). Unfortunately, racial-ethnic minority caregivers of children with autism (CCA) are less likely to contact a physician or healthcare professionals about their concerns and more likely to delay their contact to have their child evaluated (Zeleke et al., *Journal of Autism and Developmental Disorders 49*(10), 4320–4331, 2019). However, little evidence exists to explain why such a gap exists in the help-seeking behaviors between White and racial-ethnic minority CCA. To address this knowledge gap, we conducted a systematic literature review to identify articles that have studied barriers in help-seeking for racial-ethnic minority CCA. A broad literature search across four databases was conducted (i.e., PubMed, PsycINFO, Education Resources Information Center, and Child Development and Adolescent Studies). The coding team identified 17 articles on help-seeking barriers for racial-ethnic minority CCA. A thematic analysis was used to narratively synthesize the help-seeking barriers identified across these 17 studies. Four themes emerged from our findings: logistical barriers, provider competence, ASD literacy, and cultural stigma. We also provided clinical recommendations for healthcare providers working with families with racial-ethnic minority CCA.

## A Systematic Review of Help-Seeking Barriers for Racial-Ethnic Minority Caregivers Accessing Diagnostic and Autism Intervention

Autism spectrum disorder (ASD) affects about 1 in 36 children aged 3–17 in the USA [45], with an estimated lifetime cost of $2.4 billion for education and healthcare services [11]. Despite this, significant disparities persist in the diagnosis and treatment of ASD, particularly among racial-ethnic minority children [15], Maenner, 2023; [74]. These children are more likely to experience a median delay of 6 months in receiving an ASD diagnosis, which limits access to early interventions crucial for improving outcomes prognosis [15, 19, 72, 74]. This is particularly concerning as racial-ethnic minority children are now the numeric majority in the USA (Vespa et al., 2020); yet, there is limited research to guide targeted intervention efforts particularly for children who experience delayed diagnoses, which provides specialized support based on an child’s specific needs, such as specific autism interventions such as Applied Behavioral Analysis (ABA) services as well as early intervention programs to increase social, language, and cognitive development. Barriers to timely diagnosis may therefore compound disparities in early intervention access. Early intervention services are important as they aim to support developmental progress as early as possible in a child’s life.

Families of children with autism often encounter significant barriers to accessing services, including long wait times for diagnosis [32]. Low-income families face additional challenges, such as delayed screenings during pediatric visits [57], and rural families must travel longer distances to receive care [48]. These obstacles are even more pronounced for racial-ethnic minority populations who are disproportionately affected by income inequality [53].

Parents are critical in seeking early interventions for children with ASD, which could help address the racial disparities highlighted in the literature [5]. A national survey shows similar reported treatment needs regardless of race or ethnicity [79]. As a consequence or in addition to delayed diagnosis, there is less access and utilization of early intervention programs like Applied Behavioral Analysis (ABA). Racial-ethnic minority caregivers are less likely to contact a physician or healthcare provider about their concerns (Magana et al., 2013; [76]).

In contrast, racial-ethnic minority CCA were less likely to contact a physician or healthcare professionals about their concerns and more likely to delay their contact to have the child evaluated. However, little evidence exists to explain why such a gap exists in the help-seeking behaviors of White versus racial-ethnic minority CCA. Health utilization research with racial-ethnic minority individuals suggests many individual and systemic factors may contribute to this gap. For example, racial-ethnic minority individuals may have lower mental health literacy; they may not understand what ASD symptoms are and when and where to seek help [43]. In addition, racial-ethnic minority CCA may not attribute ASD symptoms to a particular disorder, instead attributing the delay in a child’s language acquisition or social skills resulting from the child’s temperament [47]. Racial-ethnic minority CCA may also face systemic barriers such as a lack of culturally and linguistically competent providers, questionable trust in the healthcare systems, experiences of discrimination, and stigma of developmental disorders within their cultural communities [8, 75].

Racial-ethnic minority individuals reported more distrust in their general primary care provider than their White counterparts [36]. These systemic barriers manifest differently across racial and ethnic groups. For example, Latino caregivers often cite language barriers and fear of deportation as obstacles to seeking care [79], whereas African American families report provider bias and a history of medical mistrust as significant deterrents to early autism evaluation [12]. Additionally, Asian American families may experience heightened stigma due to cultural values emphasizing family reputation and concerns about social consequences [38], while Native American communities face unique access barriers linked to rural healthcare shortages and historical trauma [59].

To expand on these findings that address individual and systemic barriers to ASD diagnosis and intervention services, this study is grounded in the Conceptual Model of Treatment Initiation (MTI), which is based on Andersen’s health utilization model [1] and tailored for racial-ethnic minority populations. Turner et al. [71] adapted Andersen’s model by incorporating findings through conducting a literature review focused on help-seeking behaviors, mental health treatment initiation, stigma, and disparities among Asian American, Latino American, African American, and American Indian/Alaska Native populations, allowing for a more culturally nuanced analysis of barriers to care. The MTI identifies four key factors that influence access to treatment: accessibility, availability, appropriateness, and acceptability. First, accessibility includes structural barriers that can impede one’s ability to physically access treatment, such as a lack of transportation or physical clinics within a geographic region. In the context of ASD, this may be the lack of available ASD treatment providers within the family’s region. Second, availability refers to a lack of culturally competent service providers or providers of diverse backgrounds. For example, the absence of culturally adapted social skills training for children with ASD highlights this barrier [21]. Third, appropriateness captures how cultural groups conceptualize mental healthcare and the implications of treatment. Appropriateness within the context of ASD may be the lack of mental health literacy around what the various types of treatment ASD entail. Finally, acceptability captures the difficulties racial-ethnic minorities may have accepting the need for treatment and a fear that the mental health community or cultural communities will misunderstand them. For racial-ethnic minority families, this may be the fear of stigma they may receive from their cultural communities regarding ASD [71]. Built upon MTI, the present study addresses the research question: What are racial-ethnic CCA perceived barriers to accessing ASD diagnosis and intervention services?

### Help-Seeking Defined

This study reviewed research on barriers faced by racial-ethnic minority CCA in help-seeking. Rickwood and Thomas [55] define help-seeking as an adaptive coping process aimed at obtaining external assistance to address mental health concerns, encompassing both formal (e.g., health services) and informal (e.g., support from friends and family) sources. For this study, help-seeking is limited to formal healthcare services within the USA, as an ASD diagnosis is typically required to access essential services that address impairments in functioning at home and school. The study specifically explores barriers to accessing formal healthcare services, including Applied Behavioral Analysis (ABA), pediatric care, physical therapy, speech therapy, dental care, and occupational therapy. Our study also includes diagnostic services which may be provided by psychology and medical providers as well. These services are the focus of our systematic review.

The objective of this review is to systematically examine research on barriers to autism-related help-seeking among racial-ethnic minority caregivers of children with autism (CCA), which may contribute to disparities in diagnosis and subsequent interventions. Unlike previous systematic reviews (e.g., [2, 6, 62, 70]), this study focuses exclusively on families with children, as opposed to Singh and Bunyak’s [62] review, which included adults with autism. We focused on children because autism diagnosis and intervention practices have evolved significantly over the past two decades, and service systems, caregiver roles, and support needs differ markedly between childhood and adulthood.

## Methods

We employed the search and selection strategy defined by Page et al. [50] for data extraction, using PRISMA standards for reporting [50]. A broad literature search was conducted across four major databases: PubMed, PsycINFO, Education Resources Information Center (ERIC), and Child Development and Adolescent Studies. PubMed and PsycINFO were chosen due to their relevance to healthcare barriers in ASD, while ERIC and Child Development and Adolescent Studies were selected for their focus on barriers affecting children.

We carefully selected relevant search terms for the three key concepts: autism spectrum disorder (e.g., “autism,” “Child Development Disorders, Pervasive,” and “Asperger*”), racial-ethnic minorities (e.g., “minorit*”, “ethnic*”, and “racial”), and help-seeking behaviors. (e.g., “help seek*”, “healthcare” AND “disparities”, and “social services”). Only peer-reviewed articles were included; dissertations, theses, and unpublished manuscripts were excluded. An open-access systematic review protocol was published on the Open Science Framework before the search began [42].

Our research team consisted of an undergraduate research assistant, a Ph.D. candidate, a faculty member in health service psychology, and a librarian specializing in systematic reviews and psychological science literature. The Ph.D. candidate defined the scope of the review, and the librarian conducted the literature search. The Ph.D. candidate and the research assistant reviewed all articles and performed a thematic analysis. Any disagreements during article inclusion or data analysis were resolved through consultation with the faculty member.

### Study Selection

The initial search identified 358 articles. Each was reviewed based on the following inclusion criteria: (1) focus on children under 18 years of age with ASD, (2) focus on formal intervention services for ASD, and (3) examination of help-seeking barriers among racial-ethnic minority families. We excluded studies that (1) only addressed racial disparities in ASD prevalence and treatment gaps without examining help-seeking behaviors, (2) were unrelated to US healthcare services (e.g., special education services or non-U.S. medical services), (3) did not focus directly on the child (e.g., caregiver therapy), (4) were not specific to ASD (e.g., general developmental disabilities), or (5) were not original research.

**Fig. 1:**
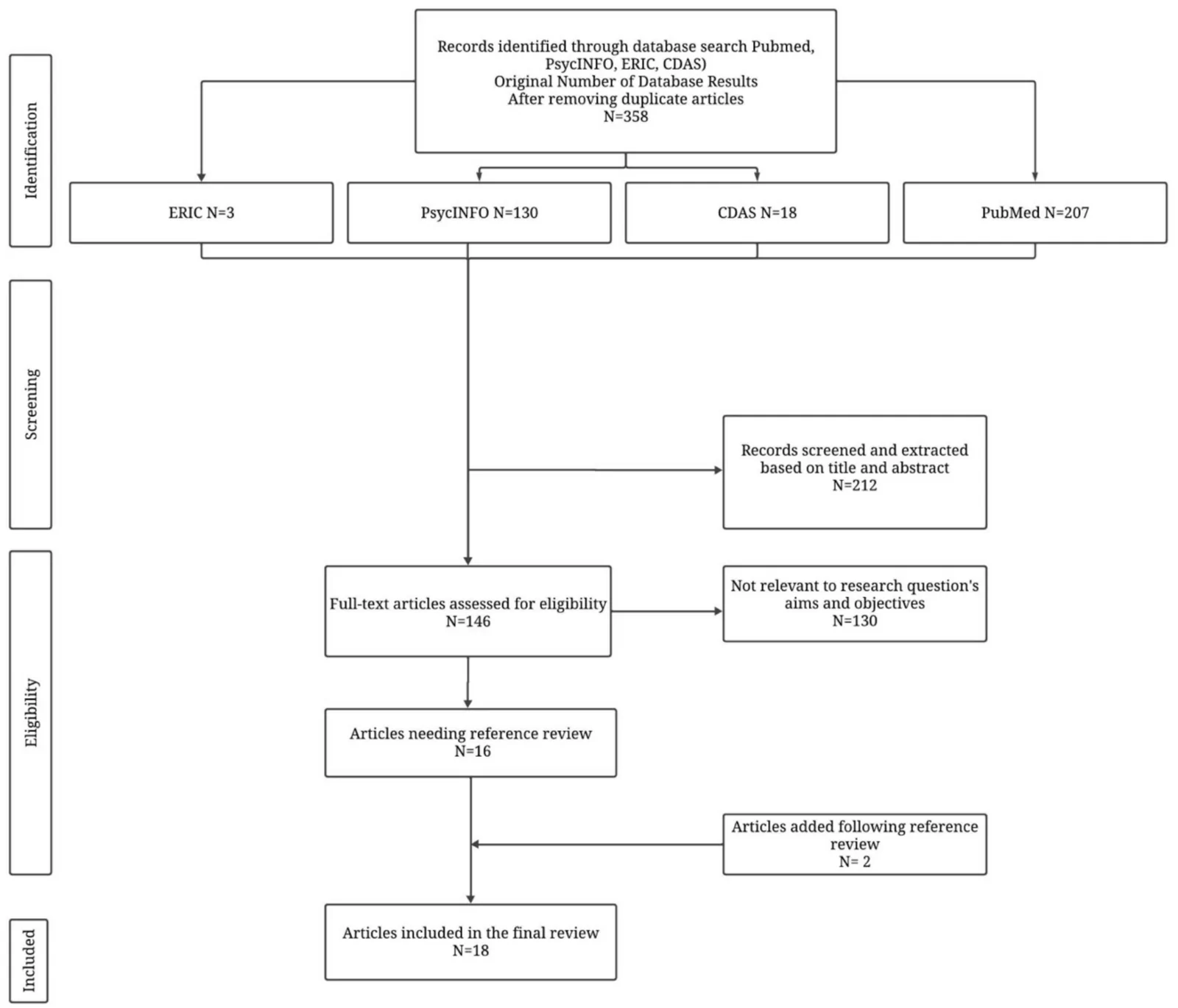
Flowchart of article selections

After a title and abstract scan, 212 studies were excluded, primarily because they were not related to racial-ethnic minorities, autism, or help-seeking barriers. The remaining 146 articles were moved to a citation management folder for full-text analysis. Of these, 130 were excluded for focusing solely on health disparities within ASD without addressing barriers for racial-ethnic minority families in accessing diagnostic and intervention services. This resulted in a selection of 16 studies. Disagreements were resolved by consensus within the research team. The final step was to review the final 16 articles to conduct a reference review to assess if any of the references in the articles should be included. Following the reference review, two additional articles were included resulting in a selection of 18 final studies (see Fig. 1 for details).

### Data Extraction and Reporting

For the final set of studies, the research team extracted key methodological characteristics, including publication year, methodological approach (e.g., quantitative, qualitative, and mixed methods), sample characteristics, and help-seeking barriers that hindered ASD diagnostic and intervention services for racial-ethnic minority families. The following sections synthesize these barriers related to help-seeking among racial-ethnic minority caregivers seeking autism diagnostic and intervention services.

### Data Analysis

We conducted thematic analyses to identify patterns that emerged from the help-seeking barriers identified in the empirical literature. Using thematic analysis following Braun and Clarke’s [10] framework provided a structured approach to be able to identify patterns across qualitative studies of shared themes and unique cultural nuances in help-seeking behaviors. This process involved systematically reading the selected articles and tabulating the identified barriers [9, 27]. Similar to analyzing primary qualitative data, thematic analysis involves coding the data and generating descriptive themes [49]. This approach is a critical method to gather key themes and findings from research to find what the help-seeking barriers are in each study [10, 33]. The approach taken to analyze the selected articles was a hybrid of inductive and deductive coding [26]. We took an inductive approach as the themes generated are strongly linked to the data (included research studies) and also deductive coding, given we used the MTI model [71] to inform our research study (deductive coding).

The research team carefully read and re-read the articles and identified help-seeking barriers within each study and then entered the findings into a data management system. In this step, the team read the entire research article but specifically extracted data in the results and discussion section of articles and extracted data of barriers reported by racial minority CCA. We generated initial codes by systematically identifying findings of all included studies each study and ensuring consistency between coders. The focus of data extraction was to identify novel findings from the research article being analyzed. In the second step, we gave a descriptive theme to each help-seeking barrier. The research team each grouped descriptive themes into a hierarchical structure based on similarities and differences between codes, where broader categories encompassed more specific barriers that shared common features.

The third and final step involved generating analytical themes to best describe each theme. In this step, we identified four common barriers evident across the articles to divide into help-seeking barrier themes listed in the results section of the manuscript. After we established a list of themes, we then further synthesized subthemes. When grouping themes, coders discussed which groupings applied to the MTI model [71], what categories were missing, as well as what aspects may need to be adapted. Author 1 and author 3 coded articles, dividing the work between them. A coding guide was developed to standardize the process, outlining criteria for inclusion (e.g., studies focused on help-seeking barriers for racial-ethnic minority caregivers of children under 18 with ASD in the USA) and exclusion (e.g., non-US samples, non-English articles, non-peer-reviewed articles, studies not involving racial-ethnic minority families, or caregivers of children over 18). If there was uncertainty about whether an article should be included, the team discussed it. If consensus could not be reached, an auditor—author 4—was consulted. The auditor helped resolve disagreements and reviewed the final list of included and excluded articles. Since each coder worked on different articles, interrater reliability was not calculated. To enhance trustworthiness, we engaged in regular team discussions including peer debriefing to reflect on our analytic decisions and reduce potential bias.

## Results

Table 1 summarizes the 18 studies included in the review, which comprised 13 qualitative studies, one quantitative study, three mixed-methods studies, and one that combined archival and qualitative components. In terms of the racial ethnic demographics of participants in each study, 7 articles (38.89%) focused exclusively on Black or African American caregivers, 5 articles (27.78%) focused on Latinx or Hispanic caregivers, 3 articles (16.67%) had a mix of ethnic racial minority caregivers, 2 articles (11.11%) focused on Asian American caregivers, and 1 (5.56%) article on Middle Eastern North African caregivers. Although there were no restrictions on the publication date, all articles were published between November 2010 and July 2021, with the search completed by July 2021. Sample sizes ranged from 6 to 352 caregivers. The table also details the help-seeking barriers identified in each article. We identified four common barriers: structural barriers, provider competence, ASD literacy, and cultural stigma, each of which is discussed in more depth below.

**Table 1:** Characteristics of included studies

Study	Sample	Data source	Theme	Method	Description of barriers
[3]	20 Latinx parents (11 families) from Los Angeles County; at least one parent must be bilingual (only 6 families included in results due to attrition)	Department of Developmental Services (DDS) regional center	Structural barriers—accessibility	Longitudinal qualitative	ABA therapy modifications requested from parents to continue ABA due to needing to accommodate work schedule
[4]	65 professionals; African American parents (22 mothers and 15 fathers and stepfathers; 17 extended family members); Los Angeles County; Diverse SES and education sample	DDS regional center, university hospital, center for developmental disabilities	Structural barriers—accessibility	Qualitative and archival review	Health records inconsistency overtime, being lost, or changing based on varying provider opinion
[7]	15 Latinx parents; Southern California	ASD clinics; parent support groups; word of mouth	Structural barriers—language barriers; cultural stigma; ASD literacy	Qualitative	Postponing receiving diagnosis in case the child’s behaviors fix itself; not understanding diagnosis; language barriers
[13]	8 families for a total of 24 family members (urban African Americans); 28 ASD professionals	Developmental clinic in a large Midwest hospital	Provider competence—trust in provider Cultural stigma	Qualitative	Perceived distrust with providers due to child welfare experiences; lack of acceptance and support of disability in cultural community
[18]	55 caregivers of young children diagnosed with ASD; 38 White, 9 American Indians, 3 Black/African Americans, 1 Asian, 18 Hispanic; North Carolina Urban/Rural	Autism Society of NC (ASNC)	Provider competence—culturally competent diagnosis; cultural stigma; structural barriers	Qualitative	Initial misguidance/diagnosis; mishandling of feedback sessions; lack of provider knowledge where to find resources/what is available to them; stigma and limited ASD knowledge; limited availability of services; limited Spanish-speaking professionals; wait for evaluations and limited services
[20]	20 African American/Black, 1 White, 1 Hispanic/Latino; all Female; Baltimore Area	ASD clinics; community-based organizations; family support groups; social media	Provider competence—provider discrimination; cultural stigma; structural barriers	Qualitative	Providers delay referral for evaluations or interventions; suspicion that lack of referrals is due to racism; disruptions in primary caregiver arrangements to follow up on referrals and evaluations; stigma of label from community and family members
[31]	20 mothers (3 Asian, 2 African American/Black, 8 Latina/Hispanic, 6 White/Caucasian, 1 more than one); Boston Area	ASD organizations including parent support groups, community listserv, and service agencies	ASD literacy	Qualitative	Challenges to see signs of ASD in their own children; lack of understanding of ASD symptoms and etiology
[35]	20 Arab Americans (both Muslim and Christian); 15 mothers and 5 fathers; sample across USA	Listservs; parent blogs; social media sites	Cultural stigma	Mixed methods	Need to distance from cultural community due to ASD stigma
[37]	21 single, African American/Black female caregivers (16 mothers; 5 grandmothers) with Medicaid Insurance; Atlanta Area	Autism clinic within a children’s hospital in Atlanta	Structural barriers—accessibility	Qualitative	Challenges navigating Medicaid benefits and provider accepting Medicaid; multiple caregiving and provider roles of single mothers exacerbate barriers
[38]	Parents of 3 families; South Asian and Muslim South Asia (Pakistan and Bangladesh)	Early childhood special education programs	Provider competence—provider discrimination; cultural stigma	Qualitative	Distrust of professionals who did not take in families’ cultural background; ASD professionals who appeared unconfident; not feeling as if parents could connect with other parenting support group experiences because of cultural background
[44]	413 immigrant families, 5411 nonimmigrant families; > 70% of the children from immigrant households were Hispanic	National Survey of Children’s Health	Provider competence—provider discrimination structural barriers—accessibility	Quantitative	Physicians not spending enough time with family; lack of insurance coverage; need for additional help coordinating ASD interventions and recourses
[51]	11 African American/Black mothers	Parent training and information centers; community agencies; Easterseals	Structural barriers—accessibility; provider competence; ASD literacy; cultural stigma	Qualitative	Challenges navigating services and location of services; dismissal of diagnosis from extended family; lack of knowledge of ASD; limited knowledge of physician knowledge of ASD and physician dismissal of ASD
[52]	100% of parents are African American; mix of races between professionals; 9 professionals; 11 parents; Midwest State	Community agencies; healthcare agencies; social networks; schools	Cultural stigma; provider competence—provider discrimination; culturally competent diagnosis; structural barriers—accessibility	Qualitative	Denial of their child having ASD; providers dismissing concerns for ASD because of lack of knowledge; cultural differences in managing challenging behavior; lack of accessible quality providers
[58]	16 Chinese immigrant parents; (14 mothers, 2 fathers); providers 16—7 nurse practitioners/physicians	Large academic medical center in Northeastern region of USA; support groups for parents of children with autism in Chinatown	Structural barriers—accessibility and language barriers; provider competence—provider trust; cultural stigma	Qualitative	Insurance and service access; denial of ASD diagnosis; caregiver and extended family stigma of ASD; rapport with providers; language barriers
[64]	Latino/Hispanic, Black or Korean parents (50 mothers, 6 fathers), legal guardians (2); focus groups were offered in Korean, Spanish, and English	Treatment clinics; parent meetings; social media; community settings	Structural barriers; provider competence—provider discrimination; cultural stigma	Qualitative	Providers invalidating concerns; perceived discrimination; denial of referrals; challenges coordinating work, childcare, and therapy; limited information on ASD due to language barriers; limited services in area and limited transportation; shame and embarrassment due to ASD stigma in community; delayed acceptance of ASD diagnosis
[73]	Mostly Black; Atlanta Area; 64% at or below the 2018 poverty line	Community-based autism clinic in Atlanta, GA	Structural barriers—accessibility; cultural stigma	Mixed methods	Social and economic situations caregivers faced also made it more challenging to coordinate care; caregiver self-care needed to prevent caregiving burnout to continue to care for child
[78]	352 Latinx and non-Latinx White parents	ASD clinics in Los Angeles, California; Denver, Colorado; and Portland, Oregon who are/were members of Autism Speaks Autism Treatment Network	Structural barriers—accessibility; ASD literacy	Mixed methods	Confusion about how to get help; lack of information about ASD; reluctance to ask for help for due to immigration status
[79]	33 parents of Latino children; Oregon; primarily mothers, Spanish-speaking, less than a high school education	University autism clinic research registry, county development disability service, and community advocacy organization	ASD literacy; structural barriers—language barrier; provider competence	Qualitative	Lack of info; mental health or disability stigma; machismo; limited English proficiency; poverty; provider dismisses concerns, process is complex and slow; process is inconvenient; process is uncomfortable for child

### Structural Barriers

This theme highlights the logistical and language challenges faced by racial-ethnic minority caregivers in accessing ASD services, identified in 13 studies. Two subthemes emerged: accessibility and language barriers.

#### Accessibility

Caregivers reported difficulties in locating and accessing ASD services due to various logistical issues, including a limited number of providers, long waitlists, and challenges with insurance coverage and affordable childcare. Specialty providers, such as speech therapists, were hard to find, and once located, wait times for evaluations could range from a few months to 1.5 years [18, 58]. Even when appointments were secured, caregivers often felt that providers did not spend enough time with them to deliver adequate care [44]. For families involved in the legal system, caregiving transitions, such as those due to the child welfare system or parental incarceration, made accessing services especially difficult, particularly for interim caregivers not listed as legal guardians [20].

Health insurance coverage challenges were also significant, with some insurance plans not covering recommended services [58]. Particularly for families with Medicaid insurance, locating providers who accepted Medicaid was limited [37]. Immigrant caregivers were also more likely to lack health insurance coverage compared to non-immigrant caregivers [44]. Moreover, single caregivers faced difficulties in finding affordable childcare, often leading them to adjust work schedules or their child’s therapy hours [3, 37, 58, 64]. Navigating services was further complicated by fragmented health records, where lost or inaccessible medical records delayed treatment and sometimes required re-diagnosis of ASD to continue care [4].

#### Language Barriers

Non-native English-speaking caregivers faced significant challenges in navigating the healthcare system. Several studies found that language barriers often interfered with accessing ASD treatment, receiving information on ASD, what ASD interventions and diagnostics entail, and building rapport with providers [58, 79]. Non-native English-speaking caregivers were found to have limited access to information on ASD and ASD treatment in their native language [64, 79]. Interventions provided in other languages as Spanish-speaking interventions were also scarce and difficult to find [18]. Despite this, providers and caregivers reported that the use of knowledgeable interpreters to facilitate communication of developmental disorders could build stronger caregiver-provider rapport among non-English speaking families with a child with ASD [58, 79]. However, having access to knowledgeable interpreters (i.e., in developmental disorders) during healthcare appointments was often difficult to access consistently [58].

### Provider Competence

The second theme that emerged, found in seven qualitative studies, was provider competence. This theme reflects parents’ concerns about inadequate services that discouraged them from seeking help. Across studies, the term “provider” referred to a range of professionals including pediatricians, psychologists, speech-language pathologists, and other therapists involved in the diagnostic or early intervention process. Three subthemes were identified: culturally competent diagnosis, perceived discrimination, and trust in providers.

#### Culturally Competent Diagnosis

Caregivers reported that providers often lacked cultural competence when diagnosing racial-ethnic minority children with ASD. In four studies (Table 1), caregivers noted that their providers were uninformed about autism, the diagnostic criteria, and cultural nuances in ASD presentations. Some caregivers felt their concerns were dismissed and sought second opinions, sometimes having to switch providers multiple times to find one who took their concerns seriously [13, 18, 51, 79]. Caregivers who raised their concerns with their physician were given reassuring language perceived as condescending by caregivers, such as “He passed the MCHAT at age 2, so what makes you think he has autism?” [18], pg. 249). The MCHAT is a commonly used Autism screener that is given to caregivers to fill out to screen for ASD symptoms [56]. However, the MCHAT is known to be less sensitive to children of color [14, 34]. Some caregivers felt that their concerns were repeatedly ignored and therefore switched healthcare providers multiple times before finding a provider who took their concerns seriously [79]. While families reported feeling dismissed, caregivers also felt that their primary medical providers had a lack of understanding of ASD and the diagnosis criteria, hence delaying critical referrals for intervention [51, 52, 64, 79].

#### Perceived Discrimination

This subtheme describes the discrimination experienced by racial-ethnic minority caregivers from providers, reported in seven studies. Caregivers recounted specific instances of racism, where providers made negative assumptions about them or quickly dismissed their concerns [20, 38, 44, 64]. For example, a caregiver in Dababnah et al. [20] described feeling that the provider treated her differently because of her race when she asked about treatment for her child. “You assume because I’m Black I’m not smart… I would get this whole lip service with the doctor, ‘Well, you can’t do this with Medicaid,’ I said, ‘I don’t even have Medicaid. I have private insurance’” [20], p. 329). Experiences of racial discrimination from providers were explicitly reported within studies, but racial-minority caregivers reported other instances of discrimination, including instances of gender (e.g., [52] and social class (e.g., [73]) discrimination from their child’s provider. Caregivers in Dababnah et al. [20] also reported feeling dismissed due to being perceived as young or as an inexperienced caregiver by their providers (i.e., ageism).

#### Trust in Provider

A lack of trust in providers was another significant barrier. Racial-ethnic minority caregivers were more hesitant to seek help compared to their White counterparts, largely due to perceived incompetence and discrimination from providers [13, 20, 58, 64, 78]. The distrust in providers may be due to the perceived incompetence in working with racial-ethnic minority caregivers, as described earlier in our findings [13, 14]. Furthermore, perceived instances of discrimination, as previously discussed, may have impacted trust between providers and racial-ethnic minority CCA [20, 52, 64, 73]. Legal and custodial issues were noted to also disrupt trust among providers and racial-ethnic minority CCA. For example, caregivers reported a general distrust of providers due to negative experiences with the child welfare system [20]. This distrust led to a reluctance to follow up on their child’s recommendations. Likewise, some caregivers were reluctant to ask for help, out of fear that they would be required to report their immigration status to healthcare providers, who may then report them to immigration authorities [78].

### ASD Literacy

ASD literacy refers to the knowledge racial-ethnic minority families have about autism, typical child development, and how to access autism-related services. Six studies in our review identified ASD literacy as a significant barrier to accessing diagnostic and intervention services. Language barriers contribute to a severe lack of quality information about developmental disorders within these communities [18, 51, 64, 78]. For example, in one of the studies, a Latinx mother who has a child with ASD shared, “I didn’t know anything. The day they told me [that my child had autism], I didn’t know what it was.” [79] Likewise, there has been a lack of information on autism and child development within Asian and Black community groups [18, 64]. In a quantitative study, the most common help-seeking barrier identified among 148 racial-ethnic minority caregivers by both caregivers themselves and providers was “caregiver’s lack of knowledge of ASD.” [78].

In addition to parents’ self-reports, providers also described racial-ethnic minority CCA’s ASD literacy as low [7]. Providers perceived parents’ delay in accessing ASD services was due to caregivers’ lack of knowledge in child development [64]. Providers across studies reported that they needed to provide additional explanations about the positive impact of early ASD interventions across several visits for caregivers to follow through with their recommendations [52, 58, 73]. Both caregivers and providers from Stahmer et al. [64] reported that Spanish-speaking caregivers’ lack of knowledge of child development was a contributing factor that led to a delayed diagnosis of ASD and engagement with services.

#### Cultural Stigma

Cultural stigma, the most frequently reported theme across 12 studies, refers to negative cultural attitudes toward developmental disabilities like autism. This stigma often leads to increased isolation from cultural communities, as caregivers encounter resistance and negative attitudes from family members. For example, both Black and Latinx mothers reported that fathers or extended family members resisted the autism label, invalidating or downplaying concerns about the child’s development [7, 18, 20], Pearson & Meadan). Racial and ethnic minority communities often invalidated, downplayed, denied, or placed blame on caregivers for anything developmentally atypical of the child [31, 35, 64]. Throughout the articles, there was culture-specific stigma that cultural communities engaged in. For example, some South Asian Muslim participants in Jegatheesan et al. [38] reported relatives in their cultural community placed blame on the mother for the child’s ASD diagnosis during pregnancy (e.g., not adhering to religious practices or consuming foods prohibited in their religion).

In an effort to navigate raising a child with ASD within their cultural community, caregivers discussed strategies for concealing their children’s atypical behavior [7]. For example, one mother described a strategy of covering her child’s atypical behavior by tickling her child so it would appear as if her child was laughing due to her tickling [7]. Many racial-ethnic minority caregivers reported keeping their child’s ASD diagnosis a secret from the child’s grandparents to reduce feelings of loneliness and limited support [64]. Additionally, these caregivers reported family members dismissing their concerns for fear it could cause overwhelming anxiety for older relatives [64]. In a similar instance, caregivers reported that they would hide a child that behaved differently from their cultural community [79]. Consequently, racial-ethnic minority CCA were reluctant to follow up on or acknowledge early developmental delays due to stigma within their cultural community [20]. For example, Chinese immigrant CCA reported not talking about developmental issues with other Chinese caregivers who do not have children with ASD, feeling as though it would likely be easier to talk to those who do not share the same cultural background about their child’s concerns [58].

The emotional toll of cultural stigma on caregivers was significant. Three studies highlighted how a lack of self-care and decreased social connections deepened caregivers’ isolation, making it even harder to seek support [35, 38, 73]. Some caregivers expressed that having a community of other CCA from their background would help them cope with the loss of cultural connection they experienced after an autism diagnosis [38].

## Discussion

This study is aimed at identifying help-seeking barriers for racial-ethnic minority caregivers of children with autism (CCA) to inform clinical practice, diagnostic, and intervention strategies. Our systematic review revealed four common barriers: structural barriers, provider competence, ASD literacy, and cultural stigma. These themes, while distinct, often overlap. For example, language barriers can hinder trust in providers, and cultural stigma may stem from a lack of ASD literacy.

The findings highlight both shared and distinct barriers to accessing care among different racial-ethnic caregiver groups, underscoring the need to avoid treating minority groups as homogeneous. While cultural stigma emerged as a common theme across all racial-ethnic groups in the included studies (African American/Black, Hispanic/Latinx, Middle Eastern North African, and Asian American caregivers), the ways in which stigma manifested varied based on cultural context. For example, within the subtheme of perceived discrimination, three of the five studies focused exclusively on African American caregivers [20, 52, 64], suggesting that systemic racism and historical marginalization may uniquely shape their help-seeking experiences. In contrast, language barriers were only reported in studies examining Latinx and Asian American caregivers [44], highlighting linguistic access as a distinct structural challenge for these groups. The absence of language barriers in studies focused on African American caregivers suggests that different racial-ethnic groups may encounter distinct, rather than uniform, obstacles in seeking care. These differences underscore the need for a more nuanced understanding of how cultural contexts shape help-seeking behaviors.

The first theme that emerged from our review was structural healthcare barriers. Structural healthcare barriers ranged from physical accessibility to language barriers. Prior research supports our findings, showing that racial-ethnic minorities often face similar structural obstacles when seeking both healthcare and mental health services [69]. These challenges are particularly pronounced among immigrant and refugee populations, who may be less familiar with navigating the US healthcare system. The need for specialized ASD services may further exacerbate these barriers due to the complexity of the condition, high treatment costs, limited insurance coverage, and a shortage of nearby providers. While not explored in this review, the fear of deportation may be a significant structural barrier. There is some suggested evidence that the rise and fall of autism diagnoses in Hispanic immigrants reflect immigration policies [28]. Language barriers, specifically, can be mitigated by using interpreters who are well-versed in child development terminology, which can help bridge communication gaps and improve service accessibility.

The second theme, provider competence, emerged from our analysis as a significant barrier to effective care. Past research in mental health help-seeking behavior has shown that patients are more likely to trust and adhere to provider recommendations when healthcare providers demonstrate cultural sensitivity [25, 67]. Cultural competence not only fosters rapport but also empowers providers to challenge and correct cultural stigmas associated with conditions like ASD [17]. Prior research indicates that provider and patient relationships are important to delivering quality healthcare, especially when racial-ethnic minority individuals are faced with cultural barriers [63]. Our findings suggested cultural competence was also related to how providers interpret ASD and behavior scales when working with racial-ethnic minority children. There are significant differences in behavioral scales that are used in the reporting of ASD symptoms between White children and racial-ethnic minority children. For example, racial-ethnic minority children with ASD tend to score lower on Child Behavior Checklists (CBCL) and have lower odds of total problem behaviors and conduct problems compared to White children [54]. Similarly, the MCHAT is also known to be less sensitive to racial-minority children [14, 34].

ASD literacy was another critical theme, highlighting the limited knowledge among racial-ethnic minority families regarding autism, typical child development, and how to access relevant services. This finding is consistent with broader research on mental health literacy, which shows that racial-ethnic minorities often lack awareness of mental health conditions like depression and anxiety [71]. Enhancing ASD literacy within these communities could lead to earlier detection of developmental concerns and more proactive engagement with services. For instance, increasing general mental health literacy has been shown to facilitate help-seeking behavior within communities by improving the recognition of symptoms and understanding of available resources [40]. Given the critical role of early diagnostic identification and intervention in improving outcomes for children with ASD, efforts to increase ASD literacy among racial-ethnic minority caregivers are essential.

The fourth theme, cultural stigma, was the most frequently reported barrier in the literature. This stigma, characterized by negative cultural attitudes toward developmental disabilities, often leads to increased isolation for caregivers and reluctance to seek help. Previous research has demonstrated that mental health stigma is more pronounced among racial-ethnic minorities, where blame for a diagnosis is often directed at the individual [69]. This stigma is compounded by fears that disclosing a mental health condition or seeking professional help could bring shame to the family [41]. Stigma has often been reported as a barrier to accessing mental health care among racial-ethnic minorities [22, 30, 53]. Similarly, autism also holds a similar cultural stigma as other mood disorders, such as bipolar disorder [24]. However, with autism, there seems to be more acceptance compared with adult psychotic spectrum illnesses, such as schizophrenia [24]. This may be due to people being better at understanding autism compared to other disorders such as schizophrenia, as well as autism being recognized as a childhood disorder [24]. Autism being a childhood disorder may also draw more acceptance from society compared to psychotic spectrum illnesses that occur in adulthood [24]. The theme of cultural stigma can be further interpreted with the public–private stigma differentiation concept proposed by Corrigan [16]. Public stigma is the negative public perception of the use of mental health services as private stigma, also referred to as self-stigma, which is the internalization of public stigma [16]. In the case of cultural stigma within this systematic review, many findings described how racial-ethnic minority CCA isolated themselves and their child from their cultural communities due to cultural stigma (public stigma). This public stigma also delayed caregivers in seeking out formal help for their child to avoid a stigmatizing diagnosis such as ASD [76].

MTI provided a useful framework for mapping our findings. Each of the four identified themes aligns with one of the MTI’s factors that can create barriers to treatment access: structural barriers correspond to accessibility, provider competence to availability, ASD literacy to appropriateness, and cultural stigma to acceptability. However, it is important to note that the MTI model does not explicitly address the issue of perceived discrimination, which emerged as a significant barrier in our review. This gap suggests that while the MTI model is a valuable tool, it may need to be expanded or adapted to fully capture the experiences of racial-ethnic minority caregivers in the context of ASD services. Emerging research has begun to call for anti-racist frameworks in autism care that acknowledge systemic racism and historical marginalization, and future studies should consider how these broader forces shape caregiver experiences and access to services [39, 61, 66].

### Limitations and Future Directions

There are several limitations to this review. We excluded studies focused on school-based healthcare services to concentrate on the challenges within the broader US healthcare system. Our study did not assess the quality of the research findings within each study. While most of the studies included in our systematic review were qualitative, there are emerging tools to assess the quality of qualitative findings [65, 77]. By not assessing the quality of the qualitative findings in our systematic review, it is unknown how methodically rigorous the included studies are. We also did not calculate interrater reliability, which may have enhanced the rigor of our methodology. Publication bias is another concern, as studies with non-significant results are less likely to be published, potentially amplifying the barriers we identified. Finally, potential conflicts of interest in the included studies were not systematically evaluated, which could influence the reported results.

It is important to note that while this review is focused on racial ethnic minority families, cultural perceptions of autism and experiences of systemic barriers vary widely among different racial and ethnic groups which can shape both diagnostic access and treatment engagement. Latinx caregivers often face challenges related to medical mistrust and language barriers, which can delay diagnosis and services [79]. In contrast, African American families may frequently encounter provider bias and skepticism toward professional recommendations, contributing to lower service utilization [51]. Asian American caregivers may experience heightened stigma due to cultural values emphasizing family reputation and social conformity, leading to reluctance in seeking formal diagnoses [38]. Understanding these nuanced differences is essential for developing culturally responsive diagnostics and interventions that improve access and support across diverse communities.

Our review primarily focused on studies involving primary caregivers, with a few also including providers [4, 58]. Most studies centered on Black/African American and Latinx families, with limited research on Middle Eastern North African (MENA), South Asian Muslim, and Chinese immigrant caregivers. Notably, there were no studies exclusively focused on Native American and Indigenous caregivers. Future research should explore the experiences of these underrepresented groups. Additionally, many studies included mothers as primary participants, neglecting other caregivers like fathers or grandparents, who may face unique challenges [29, 79],Wagner et al., 2020; Hong & Singh, 2014). The majority of studies were qualitative, highlighting a need for more quantitative and mixed-method research on help-seeking barriers. Further research is needed to integrate qualitative insights into quantitative and mixed-method approaches, ensuring the development of more precise constructs for measuring barriers faced by racial and ethnic minorities.

### Implications

The current review found four distinct barriers racial-ethnic minority CCA experienced in receiving timely and quality ASD diagnoses and interventions. These findings have important implications for research, training, practice, and healthcare systems.

#### Research Implications

Future research on help-seeking barriers to autism intervention and diagnostics should broaden its scope to include the experiences of Native American, Middle Eastern North African (MENA), and Asian American caregivers, groups that are underrepresented in the current literature. Additionally, while most studies in this review were qualitative, there is a need for quantitative research to explore the extent to which these barriers affect help-seeking behavior across different communities. For example, creating large-scale surveys with diverse racial and ethnic samples, as well as the development of validated quantitative measures for screening and diagnosis. Given the limited number of studies in this area, future research should not only expand its focus to underrepresented racial and ethnic groups but also increase the overall volume of studies examining systemic barriers to autism diagnosis and care. Researchers should also consider piloting intervention programs aimed at reducing cultural stigma and increasing ASD literacy among racial-ethnic minority families. For instance, the Parents Taking Action (PTA) program is a peer-led intervention initially designed for Latinx families. PTA aims to increase parents’ ASD knowledge and empower them to support their children, with cultural adaptations extending its effectiveness to diverse communities, including low-income Chinese immigrant families [46].

#### Training Recommendations

Healthcare training programs, particularly for pediatric primary care providers and psychology trainees, must emphasize the development of cultural competence in ASD screening and assessment. Given the long waitlists for formal ASD evaluations, providers must be equipped to conduct preliminary screenings and make appropriate referrals. Training should address common misconceptions that affect racial-ethnic minority families, such as the myth that being raised in a multilingual household delays language development. Additionally, providers need to be trained in interpreting ASD diagnostic tools, such as the M-CHAT and ADOS-2, within the cultural contexts of the families they serve, as these tools may yield different results across diverse populations.

#### Practice Recommendations

Pediatric primary care providers, who often have the most frequent contact with children, should conduct regular, culturally sensitive screenings for developmental disorders and make timely referrals. Tools like the Screening Tool for Autism in Toddlers and Young Children (STAT) can aid in improving early detection and diagnosis. Providers should work to build strong rapport with families, offering ASD information in multiple languages and formats, including non-print materials for families with low literacy levels. Engaging extended family members in the care process can also help address cultural concerns and enhance support systems.

Given the complexities of navigating ASD services, newly diagnosed racial-ethnic minority families should be referred to enhanced case management services to help them understand and access the healthcare system. Providers should inform families about no-cost early intervention and special education programs, particularly as racial-ethnic minority families are more likely to face economic challenges. Referring families to local autism and disability agencies can further assist them in navigating insurance and government programs for ASD services.

### Systemic Healthcare Recommendations

While individual provider–client interventions are essential, broader systemic changes are necessary to address the disparities in ASD services for racial-ethnic minority families. Healthcare providers should actively promote the utilization of existing federal and state programs, such as Medicaid waivers and social security disability insurance, to support these families. Increasing the number of ASD diagnostic and treatment providers who accept Medicaid would significantly benefit underserved communities. We also acknowledge there is a lack of providers who accept Medicaid due to low reimbursement rates [60, 68]. Given this, we also recommend that Medicaid reimbursement rates increase to help encourage the number of providers who take Medicaid.

Quality ASD information should be disseminated through psychoeducation and outreach efforts in cultural communities, including religious groups and cultural centers, using a variety of languages. Additionally, efforts to destigmatize ASD within mainstream culture are critical. One effective approach is to increase the representation of racial-ethnic minority individuals with ASD in the media. Currently, much of the media portrayal of ASD focuses on White, highly intelligent males [23]. Diversifying these representations can help reshape public perceptions and reduce cultural stigma, making ASD more broadly understood and accepted within racial and ethnic minority communities.
